# Editorial

**Published:** 1844-07-13

**Authors:** 


### THE MEDICAL EXAMINER.

PHILADELPHIA, JULY 13, 1844.

### THE BULLETIN OF MEDICAL SCIENCE.

In a bibliographical notice of several late works on Materia Medica, contained in the Medical Examiner of the 15th ultimo, allusion was made to *Bell's Practical Dictionary of Materia Medica, on the Basis of Mr. Brandé,*" which we regret to find has quite ruffled our friend, the editor of the Bulletin. In speaking of the Dictionary, a "fear" was expressed that it had "*irretrievably stopped:*" and, furthermore, in the course of our notice, it was gently *hinted* that one who is in the habit of referring to his own productions as an author, should not be too ready to condemn the practice when criticising the writings of another. "Hinc illæ lachrymæ." But why could not our brother keep cool? The weather was hot, very hot, about the time when his thunder burst forth, but where was his philosophy, and his favorite *cold water?* "A tee-totaller," one who eschews "rectified alcohol," and all villainous "liqueurs," ought surely to keep cool, even although it be *his* ox that is gored.

---

### NEW YORK JOURNAL OF MEDICINE AND CHAILLY'S MIDWIFERY.

In taking upon ourselves the labour and responsibility of conducting a Medical Journal, our wish and aim has been to be useful to the profession, more particularly in this country, by collecting and spreading before such as honor us with their patronage, whatever is new, useful, and interesting. Hence, the Examiner has been, and will continue to be, in its character, truly and essentially *eclectic.* In the accomplishment of this plan, we are restricted to no "nation, tongue or people," but "scan the world over;" and whatever interest and mortified vanity may allege to the contrary, we affect truth and science, and esteem them jewels wherever found.

One prominent object with us is to present to our readers brief notices of all new medical publications, with such an account of their character and contents as will enable any one to form some estimate of their value and importance, when circumstances do not permit a personal inspection. The task, we are sensible, is a difficult one, and, for its able performance, demands extensive and varied acquirements—greater, perhaps, than we can claim to possess. But there is one qualification, and that not the least important, to which we do make claim, and that is, *independence.* We pretend not to infallibility: but so far as a rigid determination to be impartial can make us so, we yield to no one. "Fiat justitia" is our motto, and we hope never to forget it. Any other course would not be just to authors, honorable to ourselves, or satisfactory to our patrons. Still, for a strict compliance with this just rule, great self-watchfulness is necessary. We have many and warm friends in the profession; and on more than one occasion, within the brief period of our editorial duties, we have felt how difficult it is sometimes to speak the language which truth compels us to utter. But we rejoice in believing that we have no enemies, as we certainly have no antipathies, no aversions that could possibly incline us to withhold praise where our judgment dictated it to be due. It is not to be expected, however, that authors and their reviewers can always see through the same glasses. A book is to its author like a child to its parent—the veriest hunch-back—the *Gloster* in other people's eyes, is to him an *Adonis.*

We are led to make these remarks from an article in the last New York Medical Journal, (July, 1844,) in reply to our notice of Chailly's Midwifery edited by Professor Bedford, contained in the Examiner of the 4th of May last. The article is signed D. M. R., initials which the editor says will be readily recognized. The writer of the article characterizes our "criticism" as "meager and ill-omened," charges us with not having read the work, with injustice, prejudice "against every thing French," and "especially against the publications of the commercial metropolis," &c., &c. Now the only reply we have to make to all this is, that, instead of resting these grave charges on his own anonymous assertion, it would have been wiser in him to have proved them by extracts from the "criticism" itself; and not having done so, we are content that every one shall judge of the reason. There are but two charges in the course of the whole article that we should deem worthy of notice under any circumstances. The first appears to be *stereotyped* in our sister city, ready for use on all occasions—viz: "prejudice against the publications of the commercial metropolis," of which it is sufficient on the present occasion to say that, since the Examiner has been under our charge, (six months,) but *two* medical books have come to us from the press of that city; whilst of those published in Philadelphia, the notices in the Examiner have not all been laudatory, as any one may see by referring to its pages.

The other charge is that we have complained "grievously of M. Chailly and his American annotator, that they have not made suitable mention of the works of British writers, and especially that of Dr. Churchill, edited by Professor Huston!" Our answer to this is a flat denial—and we challenge the writer to the proof. Not the least reference was made in our "criticism" to "Dr. Churchill's Midwifery *edited by Professor Huston*." And how could we complain of *M. Chailly* for such an omission, when his work was written before the publication of that of Dr. Churchill, and of course, long enough before our edition of it?

If the treatise of M. Chailly had been put forth merely as a "practical work," exhibiting the peculiar views of Chailly and his master, M. P. Dubois, we should have noticed it accordingly. But, in the *remarkable* preface to the American edition, the Editor and Translator uses this language: "*I therefore offer this treatise to the student as combining all that is new and valuable in obstetric science*," &c. Taking it up in the aspect presented by the Editor, we pointed out the important fact, that it contained few or no references to the best English, German and American writers on the subject, and therefore, was not entitled to be regarded as an exposition of "all that is new and valuable in obstetrical science," and furthermore, that these deficiencies had not been supplied in the translated edition.

This defender of M. Chailly, or rather of "his American annotator," virtually admits the justice of our criticism on this point, and offers the following awkward apology, viz:

"It did not occur to the critic of the 'Medical Examiner' that it would not comport with the plan of M. Chailly to cite authorities or quote authors, whether British or American. Himself a *practical* man, and preparing for students and practitioners a brief, original and *practical* work, such a course would have been inappropriate and absurd."

Having abandoned ground so obviously untenable, this Knight of La Mancha has deemed it necessary to substitute some other which may be equally attractive to the *student*; one, indeed, well calculated to catch the eye and excite the stare of older persons. From this new position we are permitted, in fact, to behold for the first time, a "Practical Treatise" on Obstetrics! Hear him! "The superiority of M. Chailly's 'Practical Treatise' over any of his predecessors, whether British or American, may be affirmed without undervaluing either of them (!) or in the least detracting from their peculiar merits, since it is strictly and pre-eminently what it purports to be, a 'practical treatise,' *and hence occupies a field hitherto unexplored*." Practical obstetrics "hitherto unexplored!" How fortunate for mankind that M. Chailly should, even at this late day, have appeared in the world to make known to us this "field hitherto unexplored!" Puzos, Leveret, Baudelocque, Smellie, Denman, Hunter, Hamilton, Blundell, Collins, Ramsbotham, Rigby, and thousands more, and our own Dewees too, never explored this field!

Before dismissing this subject it is proper to say that the article which we have thus briefly noticed, is accompanied by a note in the New York Journal, in which the Editor justifies its publication on the ground of "imperious duty." It is manifest, however, from the length and character of the note, that the conductor of the Journal deemed the defence made by his correspondent insufficient, for he enters himself upon the task of defending Chailly and attacking us. When the reader is informed that this supplemental defence is garnished with such phrases as, "ill-natured and unjust," "prompted both by sectional jealousy and personal interest," "black bile engendered by the envy of a rival city," "violent abuse," &c., &c., he will understand why we attempt no defence of ourselves against this assault. We have no skill in such a warfare.

---

We have received from Barrington and Haswell, of Philadelphia, their re-print of the excellent work on Midwifery by Dr. Lee, and shall notice it more particularly in our next.

---

### DEATH OF DR. OTTO.

It is with deep regret that we have to announce the decease of the venerable Dr. John C. Otto. He died in this city on Wednesday, the 26th ultimo, at the house of his son-in-law, Judge Mallory, in the seventieth year of his age.

Dr. Otto lived and practised his profession in the city of Philadelphia, nearly half a century. During this long period, not only as a physician, but in all the relations of life, he maintained a spotless reputation; and it is due to his many virtues to say that, at all times and on all occasions, they secured for him the confidence and esteem of the community in which he resided. The subjoined proceedings of the College of Physicians, of which he was Vice President, fitly express the feelings of the whole profession in this city:

"At a special meeting of the College of Physicians of Philadelphia, convened on the afternoon of the 29th ultimo, for the purpose of testifying its respect for the memory of its late Fellow and Vice President, John C. Otto, M. D., the following resolutions were unanimously adopted:

"*Resolved*, That whilst we contemplate the removal from amongst us of our late venerable and respected Fellow, as the fulfilment of the law of our being, which makes it needful for man 'once to die,' we cannot but lament the loss of one whose scientific acquirements, practical abilities, and moral worth, placed him in a position of merited eminence among the Fellows of the College.

"*Resolved*, That while we deplore our own loss, and that of the profession generally, we sympathize deeply with the family of the deceased, who have been bereaved of a kind and faithful husband and parent: and we desire to tender to them an expression of our heartfelt condolence.

"*Resolved*, That a Fellow of the College be appointed to prepare a suitable record of the life and character of the deceased, to be preserved among our archives.

"*Resolved*, That the Secretary of the College be requested to transmit a copy of these proceedings to the family of the deceased, and to have the same published in the medical journals, and one or more of the daily papers. By order of the College.

"D. Francis Condie, Sec'y."

At the Annual Meeting of the College of Physicians of Philadelphia, held July 2d, 1844, the following were elected officers for the current year:

PRESIDENT,
T. T. Hewson, M. D.

VICE PRESIDENT,
Henry Neill, M. D.

CENSORS,
Geo. B. Wood, M. D., Cha's D. Meigs, M. D.,
J. Wilson Moore, M. D., Henry Bond, M. D.

SECRETARY,
D. Francis Condie, M. D.

TREASURER,
John Rodman Paul, M. D.

